# Enhanced Replication of Virulent Newcastle Disease Virus in Chicken Macrophages Is due to Polarized Activation of Cells by Inhibition of TLR7

**DOI:** 10.3389/fimmu.2018.00366

**Published:** 2018-04-04

**Authors:** Pingze Zhang, Zhuang Ding, Xinxin Liu, Yanyu Chen, Junjiao Li, Zhi Tao, Yidong Fei, Cong Xue, Jing Qian, Xueli Wang, Qingmei Li, Tobias Stoeger, Jianjun Chen, Yuhai Bi, Renfu Yin

**Affiliations:** ^1^Department of Veterinary Preventive Medicine, College of Veterinary Medicine, Jilin University, Changchun, China; ^2^College of Food Science and Engineering, Jilin University, Changchun, China; ^3^College of Animal Science and Technology, Inner Mongolia University for Nationalities, Tongliao, China; ^4^Laboratory of Animal Immunology, Henan Academy of Agricultural Sciences, Zhengzhou, China; ^5^Comprehensive Pneumology Center, Institute of Lung Biology and Disease (iLBD), Helmholtz Zentrum Muenchen, Munich, Germany; ^6^CAS Key Laboratory of Special Pathogens and Biosafety, Wuhan Institute of Virology, Chinese Academy of Sciences, Hubei, China; ^7^CAS Key Laboratory of Pathogenic Microbiology and Immunology, Institute of Microbiology, Chinese Academy of Sciences, Beijing, China

**Keywords:** chicken macrophages, newcastle disease virus, toll-like receptor 7, macrophage polarized activation, virus growth, immune response

## Abstract

Newcastle disease (ND), caused by infections with virulent strains of Newcastle disease virus (NDV), is one of the most important infectious disease affecting wild, peridomestic, and domestic birds worldwide. Vaccines constructed from live, low-virulence (lentogenic) viruses are the most accepted prevention and control strategies for combating ND in poultry across the globe. Avian macrophages are one of the first cell lines of defense against microbial infection, responding to signals in the microenvironment. Although macrophages are considered to be one of the main target cells for NDV infection *in vivo*, very little is known about the ability of NDV to infect chicken macrophages, and virulence mechanisms of NDV as well as the polarized activation patterns of macrophages and correlation with viral infection and replication. In the present study, a cell culture model (chicken bone marrow macrophage cell line HD11) and three different virulence and genotypes of NDV (including class II virulent NA-1, class II lentogenic LaSota, and class I lentogenic F55) were used to solve the above underlying questions. Our data indicated that all three NDV strains had similar replication rates during the early stages of infection. Virulent NDV titers were shown to increase compared to the other lentogenic strains, and this growth was associated with a strong upregulation of both pro-inflammatory M1-like markers/cytokines and anti-inflammatory M2-like markers/cytokines in chicken macrophages. Virulent NDV was found to block toll-like receptor (TLR) 7 expression, inducing higher expression of type I interferons in chicken macrophages at the late stage of viral infection. Only virulent NDV replication can be inhibited by pretreatment with TLR7 ligand. Overall, this study demonstrated that virulent NDV activates a M1-/M2-like mixed polarized activation of chicken macrophages by inhibition of TLR7, resulting in enhanced replication compared to lentogenic viruses.

## Introduction

Newcastle disease (ND), caused by the virulent Newcastle disease virus (NDV), is a highly contagious and fatal viral infectious disease in birds and can have devastating economic effects on global domestic poultry production. ND is listed by the World Health Organization for Animals (OIE) as a vitally important pathogen for avian species and products, in which NDV detection in a specific geographical location often leads to trade restrictions and embargoes (1). NDV was previously synonymous with avian paramyxovirus type 1 (2); however, due to changes in taxonomy is now referred to as avian avulavirus (3). NDV is an enveloped virus containing a single-stranded, negative-sense, non-segmented RNA genome that is approximately 15,000 nucleotides in length. Six structural proteins are encoded, including the nucleocapsid (N) protein, phosphoprotein (P), matrix (M) protein, fusion (F) protein, hemagglutinin-neuraminidase (HN) protein, large (L) protein, and two non-structural V and W proteins (2). Strains of NDV are grouped into virulent (velogenic), intermediate (mesogenic), and non-virulent or low virulent (lentogenic) on the basis of the clinical signs seen in infected chickens (2).

Newcastle disease virus is categorized genetically into two classes with 18 genotypes in class II and only one genotype in class I, according to phylogenetic relationships of the F gene (4). Class I NDV isolates are distributed worldwide and are isolated frequently from waterfowl, shorebirds, wild birds, and live bird markets (LBMs). All reported strains are thought to be low virulence except for one strain, chicken/Ireland/1990 (5–11). Class II NDVs are further divided into 18 genotypes (I –XVIII) (12), contain viruses that have been isolated from multiple birds. Most Class II NDV are virulent and cause devastating economic losses to the poultry production worldwide (4), while lentogenic strains may be used as vaccines.

Newcastle disease virus infection *in vivo* results in various reactions and clinical symptoms based on its pathogenicity. Even though all strains of NDV belong to one serotype and cause similar humoral immune responses, differences in host innate immune responses play a role in the resistance to ND due to genetic variation of host, virulence, and genotypes of virus (13).

The host innate immune response to virus infection is designed to limit virus replication, growth, and spread in order to give the host time to develop the virus-specific adaptive immune responses (14). The primary components of innate immunity of birds are (a) physical and chemical barriers, such as skin, epithelia, and feathers; (b) phagocytic cells, including dendritic cells, macrophages, and natural killer cells; (c) inflammatory mediators, cytokines, and complement proteins (13). Macrophages, as one of the first lines of defense against microbial infection, exert numerous biological functions across a broad spectrum of acute and chronic inflammatory conditions *via* secreting high amounts of chemokines and cytokines, orchestrating host innate and adaptive immune responses, and clearing infected and dying cells to aid recovery (15).

In response to microenvironmental signals, mammalian macrophages polarize into dynamic specialized functional pro-inflammatory M1 (classically activated macrophages) and anti-inflammatory M2 (alternatively activated macrophages, TAM) phenotypes (16–21). M1 macrophages play a vital role in virus clearance and host immune responses, but excess inflammation is harmful to tissues and organs (22). By contrast, M2 cells contribute a major role in protecting tissues and organs. The M1/M2 responses from virus infection must be balanced by inhibitory and regulatory effector mechanisms to protect bystander cell, tissue and organ damage from the effects of excess inflammation, preserve oxygenation, and promote host tissue and organ repair after viral clearance (22–25). As their mammalian counterpart, plasticity also is a hallmark of chicken macrophages, and in response to microenvironment signals, including microbial infection and pathogenesis of infectious diseases (26–36), these cells undergo different forms of polarized activation, the extremes of which may called pro-inflammatory M1-like macrophages and anti-inflammatory M2-like macrophages.

Macrophages, including chicken macrophages, partly rely on the detection of characteristics of viral nucleic acids in response to virus infection (28, 37, 38). Recognition of viral nucleic acids triggers the induction of type I interferons (IFNs) that induce macrophages into an antiviral state and activate immunoregulatory functions in nearby cells. A subset of pattern recognition receptors includes toll-like receptors (TLRs), which recognize different pathogen-associated molecular patterns (PAMPs) and induces intracellular signals responsible for the activation of genes that encode for pro-/anti- (M1-/M2-like) inflammatory chemokines and cytokines, anti-microbial peptides, and anti-apoptotic factors (28, 37, 39). There is a total of 13 known TLRs in mammals (TLR1–13), with each TLR recognizing and responding to different pathogen components (40). In birds, a total of 10 TLRs have been identified and include two isoforms each of TLR1 and TLR2, which detect triacylated, and diacylated lipopeptides. TLR3, 4, 5, and 7 detect dsRNA, LPS, flagellin, and ssRNA, respectively. TLR15 has been shown to recognize yeast proteases while TLR21, a functional homolog of mammalian TLR9, detects dsDNA (41). TLR3, 7, and 21 are located in the cytoplasm, while TLR1, 2, 4, 5, and 15 are located on the cell surface (42). Previous data demonstrated that chicken origin TLR7 can exert specific abilities against viral and bacterial infectious diseases of birds, such as avian influenza (37) and Salmonella (43).

To date, NDV-induced macrophage polarized activation and its role in anti-tumor cytotoxicity, cytokine release, and immunoregulation have been widely investigated in mice and humans (44–47). Although most reliable markers for mammalian macro-phage polarized activation are not available for chicken macrophages, chicken macrophages are similar to their mammalian counterparts since they have the capacity to change their pheno-type in response to the microenvironmental signals (35, 48). However, whether NDV has the capacity to change chicken macrophage phenotype during viral infection mainly depends on the virulence and genotypes of virus. The specifics of this phenomenon and underlying molecular mechanisms are still unclear.

In the present work, we explored the polarized activation patterns of chicken macrophages and correlation with infection and replication using three different NDV genotypes of varying virulence. Levels of M1- and M2-like polarized activation-related genes and proteins in chicken macrophage cell line HD11 were used for presentation of different forms of NDV-induced chicken macrophage polarized activation. In addition, we explored the role of chicken origin TLR7 on virus replication and chicken macrophage polarized activation caused by different virulence and genotypes NDV strains.

## Materials and Methods

### Ethical Statement

All experiments performed at the Jilin University were reviewed and approved by the Jilin University Experimental Animal Care and Use Committee.

### Cell and Viruses

Chicken origin HD11 cell (permanent chicken bone marrow macrophages cell line) was kindly provided by Prof. Daxin Peng, College of Veterinary Medicine, Yangzhou University (49). NDV commercial vaccine strain LaSota (lentogenic, genotype II within class II, GenBank: AF077761.1) was obtained from ATCC. NDV virulent strain NA-1 (genotype VII within class II, GenBank: DQ659677.1) was isolated from a goose farm in Nong’an of Jilin province, China in 1999 (50). NDV lentogenic strain F55 (Chicken/CH/JL/CC05/2015, genotype Ib within class I, GenBank: KT892749.1) was isolated from chicken in LBMs of Changchun, Jilin, China in 2015 (51). HD11 cell was cultured in DMEM/F-12 (Dulbecco’s Modified Eagle Medium/Nutrient Mixture F-12) supplemented with 10% fetal bovine serum (FBS) (Gibco, Shanghai, China), 100 µg/ml streptomycin and 100 U/ml penicillin (Gibco, Shanghai, China) at 37°C under 5% CO_2_. All NDV strains were grew in the allantoic cavity of 9- to 10-day-old specific pathogen-free (SPF) chicken embryonated eggs (MERIAL, Beijing, China) and purified directly from the allantoic fluid as described in a previous study (52).

### NDV Infection of Cells

**Figure 1:**
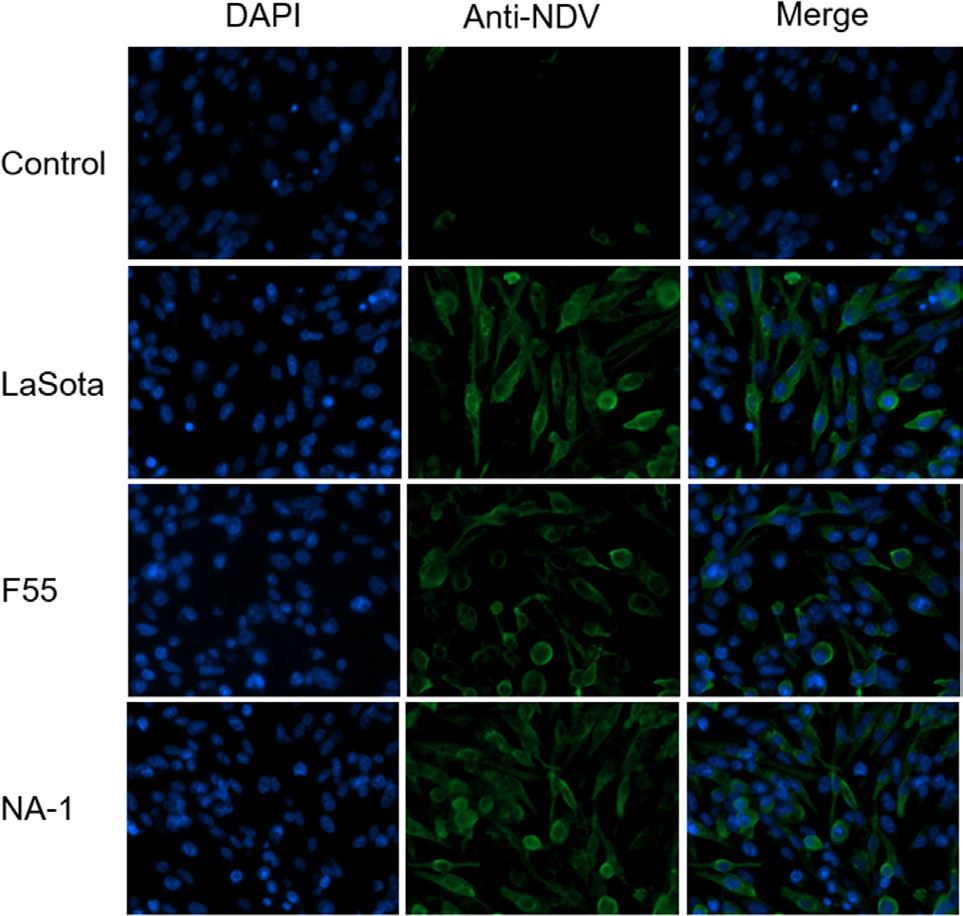
Characteristics and infection efficiency of three different virulence and genotypes Newcastle disease virus strains in chicken macrophages analysis by immunofluorescence. HD11 cells were infected with three different virulence and genotypes NDV strains (including virulent class II genotype VII strain NA-1, lentogenic class II strain genotype III strain LaSota, and lentogenic class I genotype I strain F55) at a MOI of 2 for 24 h, then viral infection efficiency was determined by immunofluorescence. Primary Mouse anti-NDV polyclonal antibody for NDV and secondary Goat Anti-Mouse IgG H&L (Alexa Fluor^®^ 488) antibody were used for immunofluorescence, and 4′,6-diamidino-2-phenylindole was used for labeling DNA to define the nuclear compartment.

HD11 cells were planted into a 24-well cell culture plate at a viable cell density (determined by Trypan blue exclusion, Sigma, Shanghai, China) of 3 × 10^5^ cells per well in complete DMEM/F12 containing 10% FBS, 100 µg/ml streptomycin, and 100 U/ml penicillin at 37°C under 5% CO_2_ for 8 h. Cells then were washed three times with phosphate-buffered saline (PBS), and supernatant was changed into fresh DMEM/F12 supplemented with 100 µg/ml streptomycin and 100 U/ml penicillin without FBS. Thereafter, cells were absorbed with virus at 2 multiplicity of infection (MOI) for 1 h and fresh medium was added into the well and then incubated with 4, 12, 24, 48, and 72 h post infection (hpi), respectively. Subsequent to infection, viral genome load and genes of target expression levels in the cells were detected by qPCR, as well as the virus titer in the supernatants was measured using a micro-HA method. For viral infection efficiency detection, HD11 cells were planted into a 6-well cell culture plate containing sterile coverslips at a viable cell density of 1 × 10^6^ cells per cell for 8 h. Cells then were rinsed three times with PBS and supernatant was changed into fresh incomplete DMEM/F12 without FBS. Thereafter, cells were absorbed with virus at 2 MOI for 1 h and fresh medium was added into the well and then incubated with 24 hpi. Subsequent to infection, cells were acetone fixed (5 min), permeabilized with 0.1% Triton X-100 for 5 min, and then incubated in 10% FBS in 0.1% PBS-Tween for 0.5 h to block non-specific protein–protein interactions. The cells were then incubated with the primary polyclonal antibody for NDV prepared by our lab (Mouse anti-NDV, 1:200) overnight at +4°C. Then cells were incubated with secondary Goat Anti-Mouse IgG H&L (Alexa Fluor^®^ 488) (ab150113) (abcom, Shanghai, Beijing) at 2 µg/ml for 1 h. 4′,6-diamidino-2-phenylindole (DAPI) was used to stain the cell nuclei (blue) at a concentration of 1.43 µM. Coverslips were inverted onto glass slides for immunofluorescence detection. Positive staining was evaluated using a laser confocal microscope (Leica, TCS SP5 Confocal Spectral Microscope Imaging System, Taipei, Taiwan). In a preliminary study, three different MOI (0.1, 2, and 10) were tested and it was verified that 2 MOI was ideal for all three NDV strains used (the infection efficiency rate of all three NDV strains ranged from 85 to 95% at 24 hpi and non-cytotoxic to cells when treated with virulent strain NA-1 early after infection from 4 to 24 hpi, data as shown in Figure 1 and Figure S1 in Supplementary Material). The negative control (100 µg/ml streptomycin and 100 U/ml penicillin but no virus was added into the same volume of NDV culture medium) were harvested following the same process.

### Macrophage Treatment with TLR7 Agonist and Cell Infection with NDV

To determine the roles of TLR7 on the replication of NDV strains in chicken macrophages *in vitro* and the status of chicken macrophage polarized activation caused by NDV infection, a TLR7 agonist loxoribine (7-allyl-7,8-dihydro-8-oxoguanosine; InvivoGen, San Diego, CA, USA) was used. Loxoribine was dissolved in DMSO at a stock concentration of 100 mM. HD11 cells were planted into a 24-well cell culture plate at a viable cell density (determined by Trypan blue exclusion, Sigma, Shanghai, China) of 3 × 10^5^ cells per well in complete DMEM/F12 containing 10% FBS, 100 µg/ml streptomycin and 100 U/ml penicillin at 37°C under 5% CO_2_ for 8 h. Subsequently, cells were pretreated with 1 mM TLR7 agonist loxoribine for 6 h and followed by infection of different genotype NDV strains at a MOI of 2 for 48 h; thereafter, both viral genome load and genes of target expression levels in the cells and the virus titer in the supernatants were measured.

### Total RNA Isolation, cDNA Synthesis, PCR, and qPCR Data Analysis

Total RNA from cells was extracted by Eastep Super Total RNA Extraction Kit (Promega, Fitchburg, MA, USA) according to the manual’s recommendations. RNA concentration and purity were evaluated by A260 and A280 measurements using a NanoDrop ND-1000 spectrophotometer. A260/A280 ratio for all RNA samples extracted spanned between 1.90 and 2.15, reflecting RNA high purity. RNA integrity was determined by the ratio of 28S/18S rRNA bands after electrophoresis in denaturing 1% agarose gel. To ensure the quality necessary for gene expression analysis, all samples extracted had a 28S/18S rRNA ratio more than 1.7.

One thousand nanograms of total RNA was reverse tran-scribed using Moloney murine leukemia virus reverse transcrip-tase (Promega, Fitchburg, MA, USA) for first strand cDNA synthesis with 5 µM random hexamer primer and 5 µg oligo-dT according to the manufacturer’s instructions. Briefly, primer and RNA were mixed and incubated at 70°C for 5 min and then cooled on ice for 5 min and followed by room temperature for 5–10 min. Then cDNA synthesis was started after adding tran-scription mixture prepared previously at 42°C lasting 1 h for reverse transcription. Finally, the reverse transcription was stopped at 70°C for 15 min. All cDNA samples were diluted 1:5 with DNase/RNase-free water and stored at −20°C for further studies.

PCR was conducted using the ABI StepOne Real-Time PCR system (Applied Biosystems, CA, USA), based on Fast Start Universal SYBR Green Master kit (Roche, Basel, Switzerland) and TransStart Probe qPCR SuperMix (TransGen, Beijing, China). The PCR mixture contained 1 µl cDNA (10 ng), 1 µl (5 µM) of each primer, 10 µl PCR mix, and DNase/RNase-free water up to a total volume of 20 µl. First, one cycle at 50°C for 2 min and 95°C for 10 min, followed by 40 cycles at 95°C for 15 s and 60°C for 90 s. PCR were performed in 48-well optical reaction plates (Sangon, Shanghai, China). To evaluate that the used primers produced only a single PCR product, a melt curve stage was added after thermocycling from 60 to 95°C by increasing 0.5°C per cycle in the SYBR Green qPCR. The primers and probe are listed in Table 1.

**Table 1:** Primers and probe of qPCR were used in the work.

Primers	Sequences (5′–3′)	Length (bp)	Reference
NDV-I	F: ACTTCGATTCTGCCTTACCAT	188	This study
	R: TTCCTCACTCCCGACCTG		
NDV-II	F: AGTGATGTGCTCGGACCTTC	121	(53)
	Probe: TTCTCTAGCAGTGGGACAGCCTGC		
	R: CCTGAGGAGAGGCATTTGCTA		
β-actin	F: CAGACATCAGGGTGTGATGG	183	(54)
	R: TCAGGGGCTACTCTCAGCTC		
HMBS	F: GGCGGCTTTGGTGACTCTAG	131	(54)
	R: ATCGAACCCTGATTCCCCGT		
iNOS	F: GGCAGCAGCGTCTCTATGACTTG	185	(55)
	R: GACTTTAGGCTGCCCAGGTTG		
IL-1β	F: TGGGCATCAAGGGCTACA	244	(56)
	R: TCGGGTTGGTTGGTGATG		
IL-10	F: GCTGCGCTTCTACACAGATG	202	(57)
	R: TCCCGTTCTCATCCATCTTC		
PPAR-γ	F: GGGCGATCTTGACAGGAA	175	(58)
	R: GCCTCCACAGAGCGAAAC		
TLR7	F: AGAGACTGGCTTCCAGGACA	218	(57)
	R: CAGCTGAACATACCGGGACT		
IFN-α	F: GACATGGCTCCCACACTACC	348	(57)
	R: AGGCGCTGTAATCGTTGTCT		
IFN-β	F: GCTCACCTCAGCATCAACAA	186	(57)
	R: GGGTGTTGAGACGTTTGGAT		

qPCR data analysis was performed in this work has been descri-bed previously (54). Briefly, relative expression of the gene of interest was analyzed by the ΔC_t_ method, where ΔCt = (C_t_ target gene, test sample—C_t_ housekeeping genes, test sample). Relative quantities of target gene was calculated as 2^−ΔCt^ ± SEM and normalized to the geometric mean of β-actin and hydroxymethyl-bilane synthase housekeeping genes in this work.

### Virus Titer Measurement

The ability of three different NDV strains (including class II virulent genotype VII strain NA-1, class II lentogenic strain genotype III strain LaSota, and class I lentogenic genotype I strain F55) to replicate and grow in chicken macrophages was investigated *in vitro*. Replication and growth kinetics of three different virulence and genotypes of NDV were assessed by multi-step growth curves in HD11 cells. Cells were seeded into 24-well cell culture plates at a viable cell density of 3 × 10^5^ cells/well and inoculated with each virus at a 2 MOI; thereafter, the viral genomic RNA load in the cell and infectious virus titer in the supernatants were determined by qPCR and micro-HA method at specific hpi, respectively.

The virus titer in the supernatants was quantified by the micro-HA method as described previously (52). Briefly, after adding an aliquot of 100 µl medium to each well, twofold dilutions of culture supernatants or virus prepared previously were transferred to the 96-well cell culture plate. Each dilution was distributed to 8 wells. Supernatants and virus were transferred in a descending manner, from the higher (2^−9^) to the lower (2^−1^) dilutions using one cell culture plate. An aliquot of 50 µl of a chicken embryo fibroblast suspension containing 10^6^ cells/mL was then added to very well and 96-well cell culture plates was incubated at 37°C under 5% CO2 for 48 h. A medium control, cell control, and virus control were also included in every cell culture plate. The virus titer was measured by the traditional HA method using a 0.5% chicken red blood cell suspension at 4°C and reading in a vertical position after 5–10 min.

### Nitrite Assay

Nitrite (a stable metabolite of nitric oxide, produced from polarized activation M1-like chicken macrophages) concentration in the cell culture supernatants was measured by the Griess assay as described previously (35). Briefly, 100 microliters of supernatants from each tested well was transferred to the 96-well ELISA plate and mixed with 50 µl of 0.1% naphthalenediamine and 50 µl of 1% sulfanilamide (both were prepared in 2.5% phosphoric acid solution). After incubation at 25°C for 10 min, the nitrite concentration was determined by measuring absorbance at 595 nm of each well in a microplate reader (Molecular Devices, Sunnyvale, CA, USA). Sodium nitrite standard solution (Sigma, Shanghai, China) also including in every ELISA plate.

### Lactate Dehydrogenase (LDH) Assay

For detection of the cytosolic enzyme LDH concentration (U/ml) in HD11 cell upon three different genotypes NDV strains infection, characteristic for membrane damaging effects, the colorimetric LDH assay kit (Nanjing Jiancheng Bioenginering Institute, Nanjing, China) was used according to the manufacturer’s instructions. Briefly, HD11 were pretreated with TLR7 agonist for 6 h and then infected with each of three different NDV strains at MOI of 2 for 48 hpi; thereafter, LDH concentration in the 30 µl undiluted cell culture supernatant was determined using an ELISA reader (Labsystems iEMS Reader, Helsinki, Finland) at a 492 nm wavelength by monitoring the reduction of NAD^+^ in the presence of lactate.

### Statistical Analysis

The results are indicated as the mean ± SEM of at least four individual samples per group (*n* = 4–6). We used analysis of variance, as calculated by Prism 5 (GraphPad Software, Inc., CA, USA), to establish the statistical significance of differences between the experimental groups. Two-tailed unpaired *t*-test with Welch’s correction was used to analyze the comparisons of individual inter-group. Differences were significantly considered at *P < 0.05, **P < 0.01, and ***P < 0.001 as compared with the respective control or vehicle groups.

## Results

### Virulent NDV Replicates Rapidly than Lentogenic Viruses in Chicken Macrophages

**Figure 2:**
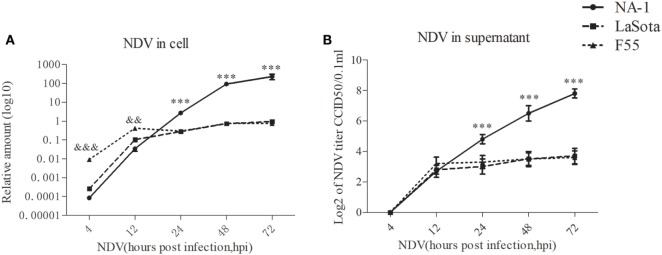
Virulent Newcastle disease virus (NDV) replicates rapidly than lentogenic viruses in chicken macrophages. The relative viral genome load in HD11 cells infected with three different virulence and genotypes NDV strains (including virulent class II genotype VII strain NA-1, lentogenic class II strain genotype III strain LaSota, and lentogenic class I genotype I strain F55) at a MOI of 2 for 4–72 h post infection (hpi) was measured by qPCR **(A)**. The virus titer in the supernatants was quantified by the micro-HA method **(B)**. The relative virus genome amount was normalized to the geometric mean of cellular endogenous genes β-actin and hydroxymethylbilane synthase and qPCR data were calculated using the 2^−ΔCt^ method. Furthermore, viral RNA genome in cells and infectious virus titer in supernatants were undetected in all uninfected cells. All values are shown as mean ± SEM (*n* = 4–6) and differences were considered significant if *P < 0.05, **P < 0.01, and ***P < 0.001 as compared to the two lentogenic virus LaSota and F55 strains infected cells, ^&&&^*P* < 0.001 as compared to the virulent virus NA-1 strain infected cells. All data are representative of at least three independent experiments.

Consistent with previous studies (59, 60), the amount of viral RNA in the cell and infectious virus titers in the supernatant significantly increased from 12 to 72 hpi in HD11 cells after infection with NDV strains (Figures 2A,B), although no infectious virus was detected in the supernatant at 4 hpi. While no differences in replication kinetics were observed between the two lentogenic NDV strains from 24 to 72 hpi, NDV strain LaSota presented a reduced ability to replicate in chicken macrophages (HD11) at 4 and 12 hpi, as evidenced by lower viral genomic RNA loads, when compared with the lentogenic NDV strain F55 (Figure 2A). In contrast with two lentogenic viruses, virulent NDV strain NA-1 presented a marked decrease in the viral RNA amount in macrophages by 12 hpi (Figure 2A), followed by a sharp increase from 24 hpi to a peak at 72 hpi, as evidenced by significant differences in replication kinetics between the virulent strain and lentogenic strains (Figure 2). Together, these results indicate that virulent NDV replicates more rapidly than lentogenic viruses in chicken macrophages *in vitro*.

### Dynamic Polarized Activation of Chicken Macrophages Induced by Virulent and Lentogenic Viruses

**Figure 3:**
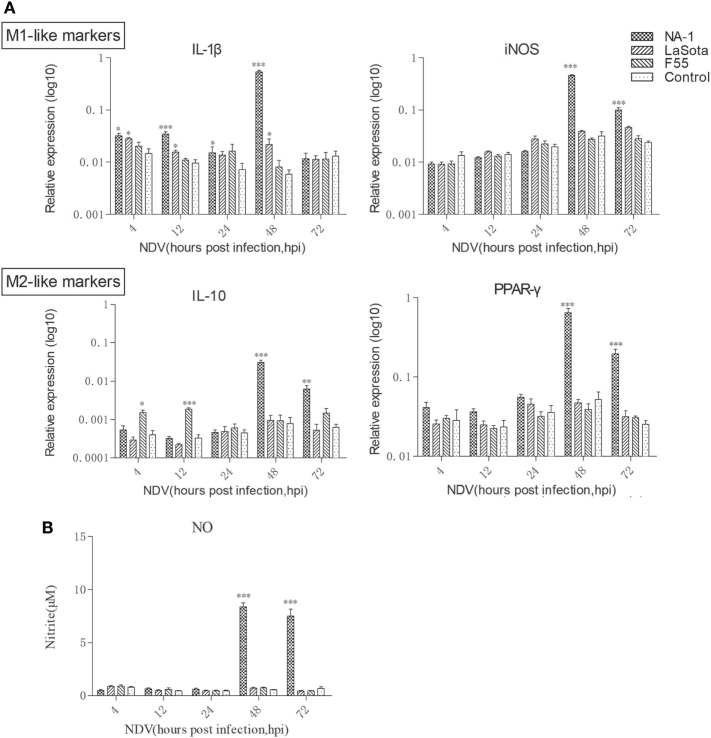
Dynamic polarized activation of chicken macrophages induced by virulent and lentogenic viruses. Expression levels of M1-like (IL-1β and iNOS) and M2 (IL-10 and PPAR-γ) genes in HD cells infected with three different virulence and genotypes Newcastle disease virus (NDV) strains at a multiplicity of infection of 2 for 4–72 h was analyzed by qPCR method **(A)**. Nitrite, a stable metabolite of nitric oxide, produced by activated M1-like macrophages was measured by the Griess assay **(B)**. All qPCR data were normalized to the geometric mean of cellular endogenous genes β-actin and hydroxymethylbilane synthase and calculated using the 2^−ΔCt^ method. All values are shown as mean ± SEM (*n* = 4–6) and differences were considered significant if *P < 0.05, **P < 0.01, and ***P < 0.001 as compared to the respective control (uninfected) cells. All data are representative of at least three independent experiments.

Compared with uninfected cells, expression of the prototypic M1- and M2-like associated genes were significantly upregulated in HD11 cells after virulent strain NA-1 infection at 48 hpi, whereas a significant change was not seen in cells infected with lentogenic viruses (Figure 3A). However, the expression pattern of these M1- and M2-like associated genes was found to be different between cells infected with the two lentogenic viruses. Specifically, M1-like associated gene IL-1β was significantly increased in cells after class II strain LaSota infection, whereas M2-like associated gene IL-10 was only upregulated in class I strain F55-infected cells (Figure 3A). Furthermore, a higher concentration of nitrite in the supernatant was only found in virulent strain NA-1-infected cells (Figure 3B). Therefore, virulent NDV infection can polarize chicken macrophages into the M1-/M2-like mixed phenotype, but the lentogenic viruses induced much more moderate polarized activation status according to their genotypes: lentogenic class II NDV infection induces a mild M1-like phenotype and lentogenic class I NDV infection causes a mild M2-like phenotype.

### Virulent NDV Blocks TLR7 Expression but Induces Higher Expression of Type I IFNs in Chicken Macrophages at the Late Stage of Viral Infection

**Figure 4:**
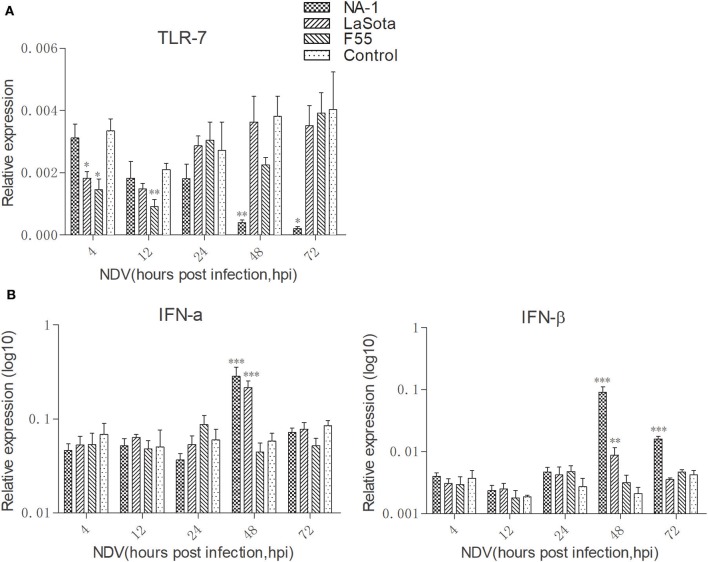
Virulent Newcastle disease virus (NDV) inhibit TLR7 expression but induce higher expression of type I interferons (IFNs) in chicken macrophages at the late stage of viral infection. Expression levels of TLR7 **(A)** and type I IFNs [IFN-α and IFN-β, **(B)**] in HD11 cells upon three different virulence and genotypes viruses for 4 to 72 h was quantified by qPCR. All qPCR data were normalized to the geometric mean of cellular endogenous genes β-actin and hydroxymethylbilane synthase and calculated using the 2^-ΔCt^ method. All values are shown as mean ± SEM (*n* = 4–6) and differences were considered significant if *P < 0.05, **P < 0.01, ***P < 0.001 as compared to the respective control (uninfected) cells. All data are representative of at least three independent experiments.

Newcastle disease virus did not significantly alter the TLR7 expression level in HD11 cells compared to uninfected cells at 24 hpi (Figure 4A). However, at 4 and 12 hpi, both class II lentogenic strain LaSota and class I lentogenic strain F55 significantly inhibited TLR7 expression in HD11 cells. By contrast, the TLR7 expression level was dramatically downregulated in cells infected with virulent NA-1 strain at 48 and 72 hpi.

The kinetics of IFN-α and IFN-β expression were then evaluated in HD11 cells after NDV infection from 4 to 72 hpi (Figure 4B). Our data clearly indicated that while class I NDV (F55) infection does not result in changes to IFN-α and IFN-β expression, class II NDV strains (LaSota and NA-1) resulted in significant increase of type I IFN expression at 48 hpi. In addition, a higher level of IFN-β expression was observed in cells infected with virulent NA-1 the late stage of virus infection (48 and 72 hpi) compared with lentogenic LaSota. Together, these results indicate that virulent NDV blocks TLR7 expression but induces higher expression of type I IFNs in chicken macrophages at the late stage of viral infection.

### TLR7-Mediated Macrophage Activation Inhibits Virulent NDV Replication and Restores Virulent Virus Induced Macrophage Polarized Activation

**Figure 5:**
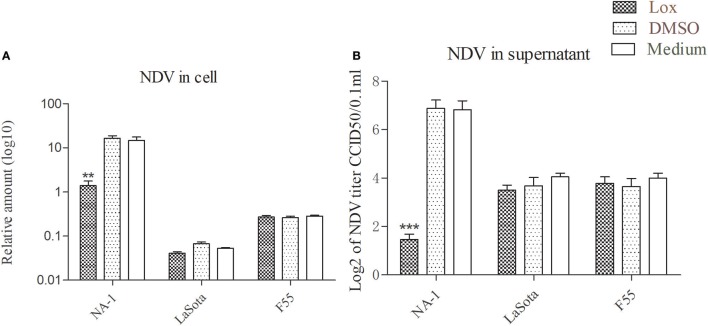
TLR7 agonist inhibit virulent Newcastle disease virus (NDV) rather than lentogenic virus replication in chicken macrophages. HD11 cells were pretreated with 1 mM loxoribine for 6 h and followed treated with three different virulence and genotypes NDV strains at 2 multiplicity of infection for 48 h; thereafter, viral genome amount in the cells and virus titer in the supernatants were measured by qPCR and micro-HA method, respectively **(A,B)**. All qPCR data were normalized to the geometric mean of cellular endogenous genes β-actin and hydroxymethylbilane synthase and calculated using the 2^−ΔCt^ method. All values are shown as mean ± SEM (*n* = 4–6) and differences were considered significant if *P < 0.05, **P < 0.01, ***P < 0.001 as compared to the respective control (untreated) cells. All data are representative of at least two independent experiments.

Consistent with previous studies (37, 43, 61), pretreatment with 1 mM loxoribine for 6 h significantly reduced the growth of virulent NDV in HD11cells as compared to the vehicle and control cells; however, such reduction was not seen in cells infected with two lentogenic viruses (Figure 5). However, cellular viability was not decreased in HD11 cells treated with 1 mM loxoribine for 6 h, as compared with untreated control cells (data as shown in Figure S2 in Supplementary Material).

**Figure 6:**
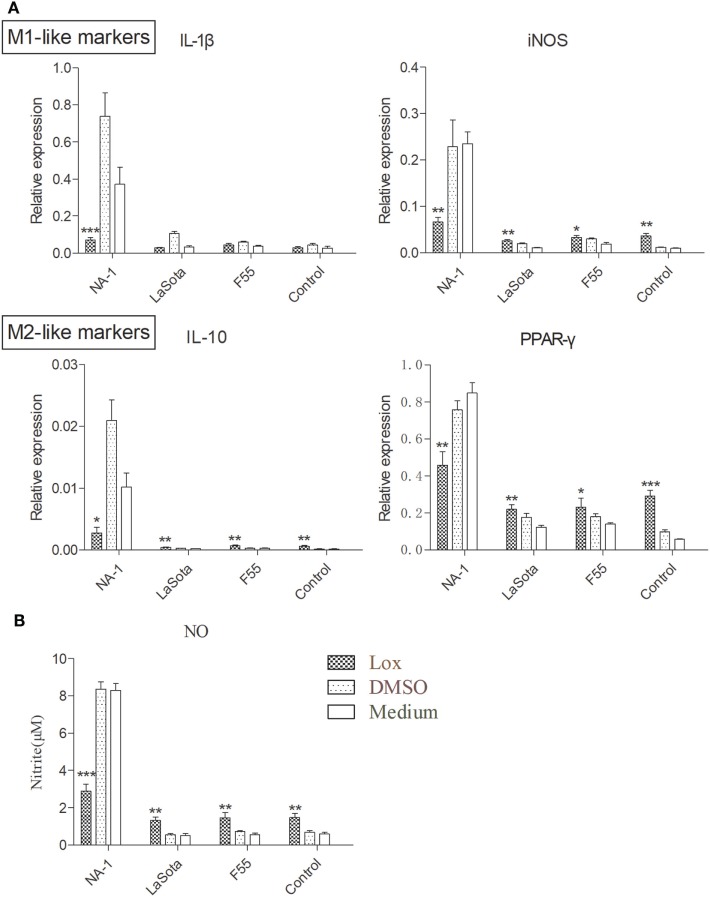
TLR7 agonist decreases macrophage polarized activation markers in cells infected virulent virus rather than lentogenic virus. HD11 cells were pretreated with 1 mM loxoribine for 6 h and subsequently infected with three different virulence and genotypes Newcastle disease virus (NDV) strains at 2 multiplicity of infection for 48 h; thereafter, expression levels of M1-like (IL-1β and iNOS) and M2 (IL-10 and PPAR-γ) genes in HD cells **(A)** and M1-like protein nitrite concentration in the supernatants **(B)** were measured by qPCR and Griess assay, respectively. All qPCR data were normalized to the geometric mean of cellular endogenous genes β-actin and hydroxymethylbilane synthase and calculated using the 2^−ΔCt^ method. All values are shown as mean ± SEM (*n* = 4–6) and differences were considered significant if *P < 0.05, **P < 0.01, and ***P < 0.001 as compared to the respective control (untreated) cells. All data are representative of at least three independent experiments.

**Figure 7:**
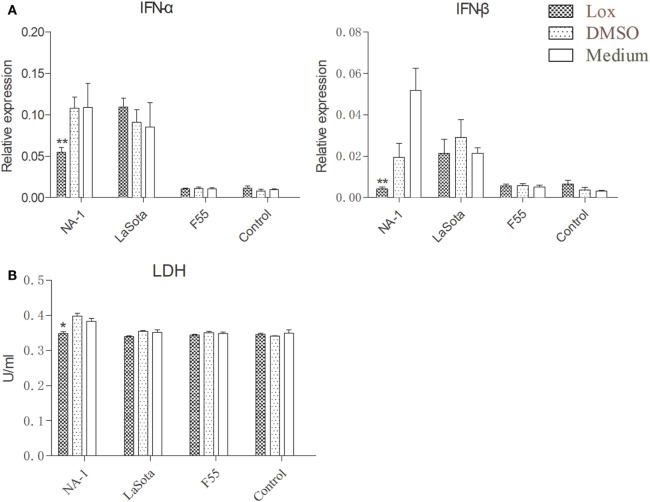
TLR7 agonist notably reduces type I interferons (IFNs) expression and alleviates cell damaging effects in chicken macrophages caused by virulent virus rather than lentogenic virus infection. HD11 cells were pretreated with 1 mM loxoribine for 6 h and subsequently infected with three different virulence and genotypes Newcastle disease virus strains at 2 multiplicity of infection for 48 h; thereafter, expression levels of type I IFNs (IFN-α and IFN-β) genes in HD cells **(A)** and lactate dehydrogenase (LDH) concentration in the supernatants **(B)** were measured by qPCR and cytotoxicity detection method, respectively. All qPCR data were normalized to the geometric mean of cellular endogenous genes β-actin and hydroxymethylbilane synthase and calculated using the 2^−ΔCt^ method. All values are shown as mean ± SEM (*n* = 4–6) and differences were considered significant if *P < 0.05, **P < 0.01, and ***P < 0.001 as compared to the respective control (untreated) cells. All data are representative of at least three independent experiments.

To further examine the effects of TLR7 agonist loxoribine on NDV-induced chicken macrophage polarized activation, HD11 cells were pretreated with 1 mM loxoribine for 6 h and then infected with three different virulence and genotypes NDV strains for 48 h. As depicted in Figure 6A, loxoribine pretreatment induced a significant increase in M1-like gene (iNOS, except IL-1β) and M2-like genes (IL-10 and PPAR-γ) in two lentogenic NDV-infected and -uninfected cells compared to the respective untreated cells. By contrast, loxoribine pretreatment resulted in a sharp decline in both M1- and M2-like genes in virulent NDV-infected cells compared to the respective untreated cells (Figure 6A), as evidenced by the concentration of nitrite in the supernatants (Figure 6B). Next, we determined that loxoribine pretreatment whether resulted in a sharp decrease in the growth of the virulent strain rather than the two lentogenic strains through inhibition of IFN-α and IFN-β. As expected, after pretreatment of HD11 cells with 1 mM loxoribine for 6 h, the expression levels of type I IFNs (IFN-α and IFN-β) were significantly decreased in cells infected with virulent virus rather than two lentogenic viruses (Figure 7A). Finally, we evaluated the cell injury caused by different genotypes NDV infection by LDH assay. As depicted in Figure 7B, pretreatment with 1 mM loxoribine for 6 h significantly decreased the virulent NDV strain NA-1 induced cell damage. Taken together, TLR7-mediated macrophage polarized activation inhibits virulent NDV replication and restores virulent virus-induced macrophage polarized activation and cell damage.

## Discussion

Macrophages play a critical role in the regulation and induction of innate and adaptive immune responses and protection of the host against pathogens (15, 62), especially viruses (19, 20, 37, 38, 63). However, recent findings demonstrated that macrophages could be a double-edged sword in virus clearance and pathology: they not only help fight against virus infection, but may also contribute to virus production and dissemination during viral infections (26, 64). In mammal models, a number of viruses have been found target macrophages and impair the function of these cells (65–67). By contrast, limited information is known about the interaction between chicken viruses and chicken macrophages. For example, previous studies showed that macrophages may be acting as a main target cell for some avian viruses infection and dissemination from the respiratory tract to nearby tissues and organs, which are necessary for continuation of the virus growth cycle (27, 68–70). Although macrophages are considered to be one of the main target cells for NDV infection and growth *in vivo* (59), very little is known about the ability of NDV to infect macrophage and the mechanisms of consequent macrophage responses to virus infections. The cell culture results in the present work indicated that chicken macrophages support the replication and growth of NDV strains of varying virulence and genotypes, and the results are in agreement with previous studies (59, 71–73).

Strains of NDV are categorized into virulent (velogenic), intermediate (mesogenic), and low virulent or non-virulent (lentogenic) on the basis of their pathogenicity in SPF chickens. However, the underlying mechanisms of virulent and lentogenic NDV strains infection as well as host responses to infections of different virulence and genotypes of NDV are still largely unknown. Although all NDV strains can infect and replicate in macrophages, it remains unclear how productive infection of macrophage by different virulence and genotypes NDV strains is impaired. In this study, we selected three different virulence and genotypes of NDV (including class II virulent genotype VII strain NA-1, class II lentogenic strain genotype III strain LaSota, and class I lentogenic genotype I strain F55), processed a preliminary experiment, and found that MOI 2 was ideal for all three NDV strains used, and no cytopathic effect to cells when treated with virulent strain NA-1 during the early stage of infection (from 4 to 12 hpi).

Our study demonstrated the replication and growth rate of three different virulence and genotypes viruses in chicken macrophages and the chicken macrophages polarized activation patterns which were induced by. Results indicated that all three NDV strains had similar replication and growth rate during the early stage of infection (from 4 to 12 hpi). However, virulent NDV replication and growth rate was shown to increase sharply from 24 hpi to a peak at 72 hpi compared to two lentogenic viruses (Figure 2), and this growth was associated with a strong upregulation of both pro-inflammatory M1-like markers/cytokines and anti-inflammatory M2-like markers/cytokines in chicken macrophages (Figure 3). Therefore, such M1-/M2-like mixed macrophages polarized activation may contribute to virulent NDV replication and growth sharply during the later stage of infection. Although two lentogenic strains did not elicit stronger M1-like or M2-like markers/cytokines production, the expression pattern of these M1- and M2-like associated genes was found to be different between cells infected with the two lentogenic viruses. In details, class I lentogenic F55 induces a mild M2-like macrophage polarized activation and class II lentogenic LaSota induces a mild M1-like macrophage polarized activation (Figure 3A).

Chicken macrophages express a number of receptors for recognition of pathogens, including TLRs. TLRs bind to PAMPs derived from viral or bacterial pathogens leading to the polarized activation of macrophages (37). Although chicken origin TLR7 functions as same as mammalian TLR7 and encodes a 1047-amino-acid protein with only 62% identity to human TLR7 (74, 75). Previous results have been indicated that chicken origin TLR7 can be recognized by viral ssRNA (75, 76), which is largely released during the infections with chicken influenza virus (61). However, the inhibition of TLR7 expression levels and higher expression of type I (IFN-α and IFN-β) IFNs were observed in chicken macrophages when treated with virulent strain NA-1 during the later stage of infection (from 48 to 72 hpi) (Figure 4). This phenomenon was not observed in lentogenic viruses infected cells.

We supposed that inhibition of TLR7 may contribute to a M1-/M2-like mixed macrophages polarized activation caused by virulent strain NDV at the later stages of viral infection. Therefore, the TLR7 ligand 7-allyl-8-oxoguanosine (loxoribine) was used for determination of TLR7 roles in chicken macrophage upon NDV infections. Like as an antiviral compound against others chicken pathogens (37, 43, 61), pretreatment of HD11 cell with 1 mM loxoribine for 6 h inhibited virulent strain replication and restored virulent virus induced M1-/M2-like mixed macrophage polarized activation (Figures 5 and 6). Furthermore, TLR7 ligand loxoribine is a stimulator for M1-/M2-like mixed chicken macrophage polarized activation (Figure 6).

It has been reported that V protein of virulent strains exhibits IFNs antagonistic activity, which contributes to the viral virulence, tissue tropism, and host range (77, 78). Interestingly, in the present study, the expression of antiviral type I IFNs was significantly enhanced, rather than weakened, in chicken macrophages following the infection with virulent NDV and it was associated with the rapid replication of virus (Figures 2 and 4). However, some virus, such as virulent strain NA-1 used in this work, is a stronger stimulator for upregulated mRNA expression of type I IFNs, that means reduction in mRNA expression of type I IFNs because of reduced viral replication and growth (Figures 5 and 7). Meanwhile, pretreatment of HD11 cell with loxoribine for decreased virulent NDV caused type-I IFNs responses and alleviated virulent NDV-induced cell damage (Figure 7).

Overall, this study demonstrated that enhanced replication and growth of virulent NDV in chicken macrophages is due to M1-/M2-like mixed macrophages polarized activation of cells by inhibition of TLR7. Although no differences in replication and growth kinetics of two lentogenic NDV strains were observed *in vitro*, the lentogenic viruses induced much more moderate macrophages polarized activation status according to their genotypes. In addition, these results with the use of TLR7 ligand 7-allyl-8-oxoguanosine (loxoribine) suggest that TLR7 could be used as an antiviral potential target against the enzootic virulent NDV infection in birds.

## Author Contributions

RY, ZD and PZ designed the study, drafted the manuscript and analyzed the data. PZ, ZD, XL, YC, JL, ZT, YF, CX, JQ, XW, QL, TS, JC, YB and RY carried out experiments. All authors read and approved the final manuscript.

## Conflict of Interest Statement

The authors declare that the research was conducted in the absence of any commercial or financial relationships that could be construed as a potential conflict of interest.
