# Supplementary material for: Enhanced Replication of Virulent Newcastle Disease Virus in Chicken Macrophages Is due to Polarized Activation of Cells by Inhibition of TLR7
Source: Front Immunol. 2018 Apr 4;9:366. doi: 10.3389/fimmu.2018.00366 (PMC5893744; doi:10.3389/fimmu.2018.00366)
Supplement: Supplementary file 1 [file Data_Sheet_1.DOC]

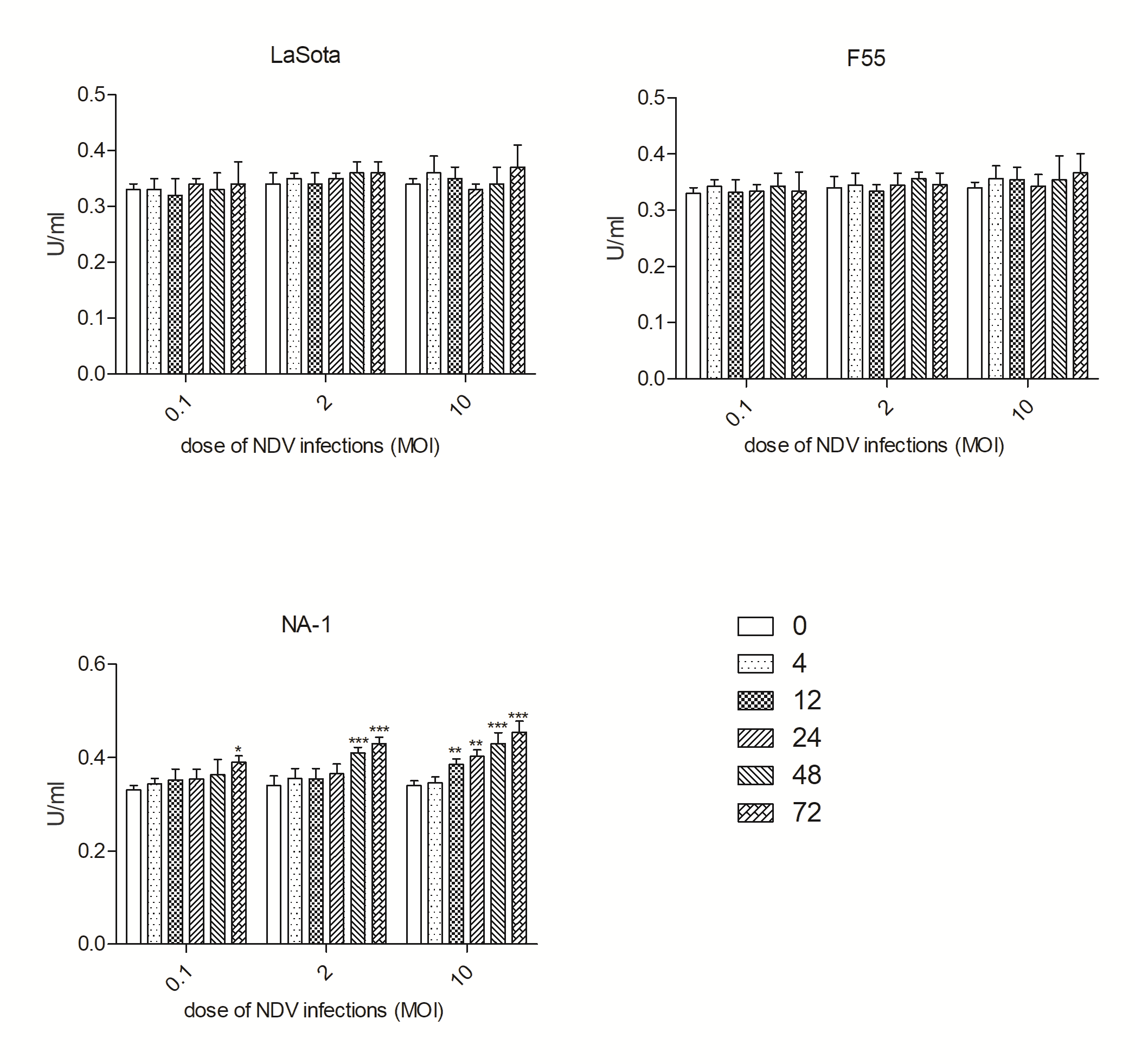


**Supplementary Figure 1. Cytotoxicity of HD11 cells infected with different virulent and genotypes NDV at 0.1, 2 and 10 MOI for 4 to 72 hours.** Lactate dehydrogenase (LDH) concentration in the supernatants were measured by cytotoxicity detection method. All values are shown as mean ± SEM (n=4-6) and differences were considered significant if *P<0.05, **P<0.01, ***P<0.001 as compared to the respective uninfected cells. All data are representative of at least three independent experiments.


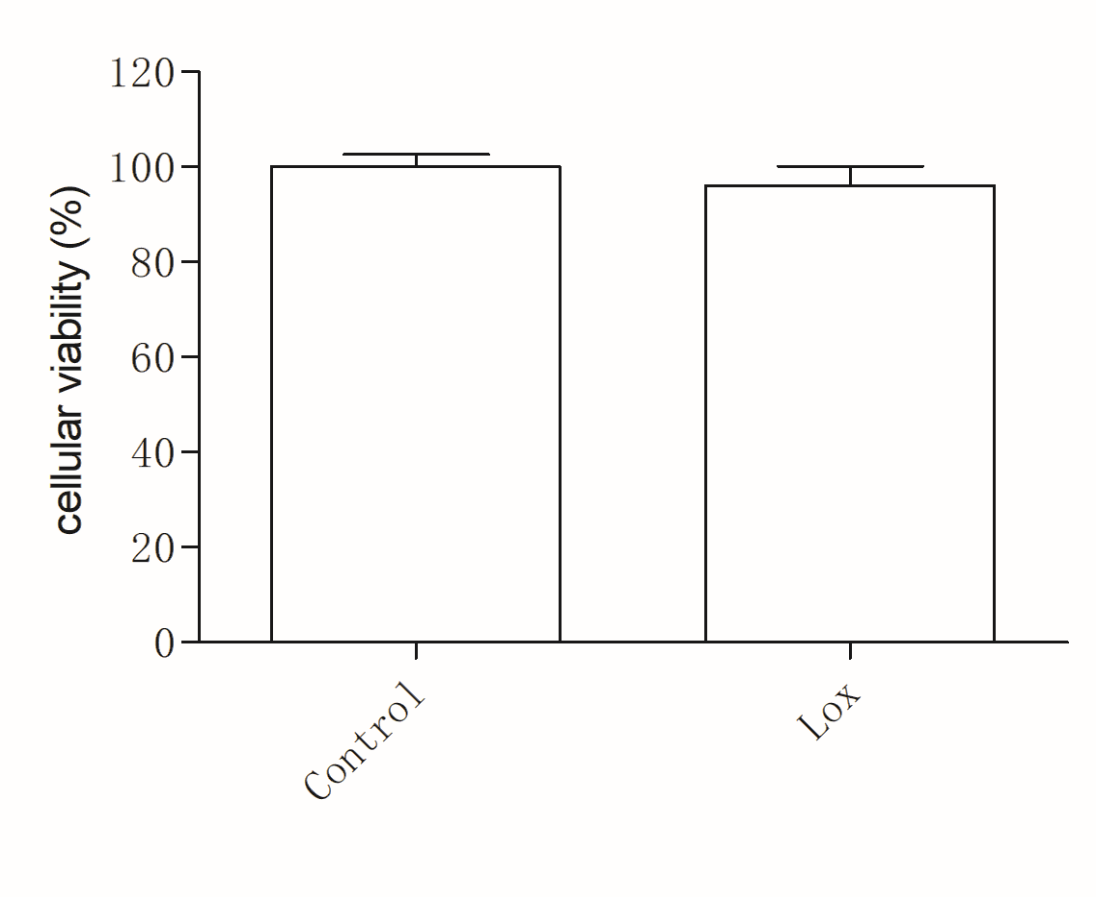


**Supplementary Figure 2. Cellular viability was not decreased in HD11 cells treated with 1mM loxoribine for 6 hours.** In brief, the cell suspension was diluted to a certain concentration and inoculated in a 96-well cell culture plate with the cell density of 5 × 10^4^ per well and six parallel wells. When cells reached 85% confluence, the cells were treated with 1 mM loxoribine for 6 hours. Thereafter, the supernatants were replaced with fresh culture medium and the cells were added with MTT solution (20 ul) (Sigma, Shanghai, China) for incubation at 37°C for 4 hours. After incubation, the MTT solution was discarded, and each well was added with 150 μL dimethyl sulfoxide, followed by shake. Subsequently, the optical density (OD) value of each well was determined at 490 nm. The experiment was repeated for three times and mean OD value was obtained. Cellular viability = (experiment group OD - blank group OD)/blank group OD.
